# Analysis of Genetic Association Between *ABCA7* Polymorphism and Alzheimer’s Disease Risk in the Southern Chinese Population

**DOI:** 10.3389/fnagi.2022.819499

**Published:** 2022-05-25

**Authors:** Lijun Wang, Yang Jiao, Aonan Zhao, Xiaomeng Xu, Guanyu Ye, Yichi Zhang, Ying Wang, Yulei Deng, Wei Xu, Jun Liu

**Affiliations:** ^1^Department of Neurology and Institute of Neurology, Ruijin Hospital, Shanghai Jiao Tong University School of Medicine, Shanghai, China; ^2^CAS Center for Excellence in Brain Science and Intelligence Technology, Ruijin Hospital, Shanghai Jiao Tong University School of Medicine, Shanghai, China

**Keywords:** Alzheimer’s disease, *ABCA7*, rs4147929, rs3764650, single nucleotide polymorphisms

## Abstract

**Objective:**

The study aimed to clarify the association of the 21 single nucleotide polymorphisms (SNPs) with Alzheimer’s disease (AD) in the population of southern China.

**Methods:**

A case-control study was conducted with a total sample size of 490 subjects (246 patients with AD and 244 age- and gender-matched healthy controls) enrolled in this study. Twenty-one selected SNPs were detected using SNaPshot assay and polymerase chain reaction (PCR) technique. Then, we assessed how these SNPs correlated with AD susceptibility.

**Results:**

The results showed that rs3764650 of *ABCA7* was closely correlated with risen AD morbidity in the allele [*P* = 0.010, odds ratio (OR) = 1.43, 95% confidence interval (CI) 1.09–1.89], dominant (*P* = 0.004, OR = 1.71, 95% CI 1.19–2.46), and additive (*P* = 0.012, OR = 1.42, 95% CI 1.08–1.86) models. However, rs4147929 of *ABCA7* was related to higher AD risk in the allele (*P* = 0.006, OR = 1.45, 95% CI 1.11–1.89), dominant (*P* = 0.012, OR = 1.59, 95% CI 1.11–2.27), and additive (*P* = 0.010, OR = 1.40, 95% CI 1.08–1.81) models. In addition, the frequencies of the G-allele at rs3764650 (*P* = 0.030) and the A-allele at rs4147929 (*P* = 0.001) in AD were statistically higher in *APOE* ε4 carriers in comparison to non-carriers.

**Conclusion:**

This study demonstrated that the G-allele at rs3764650 and the A-allele at rs4147929 appeared at higher risk for developing AD, particularly in *APOE* ε4 carriers. Moreover, it was observed that rs3764650 and rs4147929 of *ABCA7* were linked to AD. More in-depth research with a relatively large sample is needed to make the results more convincing.

## Introduction

Alzheimer’s disease (AD), an eye-catching neurodegenerative disorder in the elderly, has an increased burden dramatically on the global economic development and health care systems (Alzheimer’s Association, 2021). It is characterized by progressive deterioration of cognitive function and function impairment resulting from extracellular deposition of β-amyloid (Aβ) plaques and neuronal accumulation of neurofibrillary tangles formed by hyperphosphorylated tau protein and brain atrophy (Blennow et al., 2006; De Strooper and Karran, 2016; DeTure and Dickson, 2019; Andrews et al., 2020). Genetic factors play a crucial role in the pathogenesis of AD (Jansen et al., 2019; Kunkle et al., 2019). The most strongly and consistently associated with AD risk gene is *apolipoprotein E* (*APOE*) (Liu et al., 2013; Yamazaki et al., 2019; Serrano-Pozo et al., 2021). Previous genome-wide association studies (GWASs) have found and defined up to 20 AD susceptibility loci, including *ABCA7*, *BIN1, CLU, CR1, PICALM*, *SORL1*, and so on (Harold et al., 2009; Lambert et al., 2009, 2013; Steinberg et al., 2015; Vardarajan et al., 2015; Giri et al., 2016; Kunkle et al., 2019). Recently, studies have reported that *GRN, TMEM106B, Complement C7, RBFOX1* genes are associated with AD in various cohorts (Viswanathan et al., 2009; Lee et al., 2011; Rutherford et al., 2012; Lu et al., 2014; Jun et al., 2016; Xu et al., 2017; Kunkle et al., 2019; Hu et al., 2021). In addition, hippocampal sclerosis of aging (HS-Aging) is a common, high morbidity brain disorder that occurs in the elderly with a clinical course similar to AD. *ABCC9* and *KCNMB2* have previously been shown to be associated with HS-Aging (Nelson et al., 2014; Nho and Saykin, 2016; Katsumata et al., 2017; Dugan et al., 2021). However, most of these studies were performed statistically limited in Caucasian populations, and the inheritance of AD in other populations is relatively limited. Repeating the GWAS results in different ethnic groups can help identify SNPs that are associated with AD (Chanock et al., 2007). However, the results in other populations of European descent were inconsistent with those in the southern Chinese population. Therefore, how these candidate loci and AD in the southern Chinese population are related was still not clear. Consequently, in the present study, 21 SNPs were selected from the above studies to investigate how genes affected the AD morbidity of the southern Chinese.

## Materials and Methods

### Study Design and Participants

A sum of 246 patients with AD (137 females and 109 males, average age ± *SD*: 71.26 ± 8.46 years) were collected from September 2016 to September 2020 from the neurology outpatient clinic at Ruijin Hospital affiliated with Shanghai Jiao Tong University School of Medicine. Patients with AD dementia fulfilled the National Institute of Neurological and Communicative Disorders and Stroke–Alzheimer’s Disease and Related Disorders Association (NINCDS-ADRDA) criteria for probable AD (Dubois et al., 2007). Global cognitive abilities were measured on a scale of 0 (severely impaired) to 30 points (no impaired) using the Mini-mental Status Evaluation (MMSE) test and at least one other cognitive deficit beyond memory impairment. All participants involved were assessed by more than two neurologists with profound experience and received a couple of standard tests, including but not limited to physical examinations, medical history, and neuropsychological and neuroimaging examinations. Conversely, participants recorded with other neurological disorders, which could cause dementia were excluded (McKhann et al., 2011; Janelidze et al., 2018). So, a final total of 244 healthy controls (HC) matched for age and gender (137 females and 107 males, average age ± *SD*: 71.10 ± 8.31) were voluntarily enrolled in Shanghai, China. Healthy subjects were strictly evaluated by doctors to confirm that they have no symptoms of cognitive decline and do not comply with the criteria for mild cognitive impairment (MCI) or AD dementia (McKhann et al., 2011; Jack et al., 2018). Demographic information of the participants was presented in Table 1. This research was authorized by the Committee on Medical Ethics of Ruijin Hospital affiliated with Shanghai Jiao Tong University School of Medicine. A signed informed consent by the Declaration of Helsinki was submitted by the participants.

**TABLE 1:** Demographic and clinical characteristics of subjects included in the study.

Characteristics	AD (*n* = 246)	CN (*n* = 244)	*P-*value
Age, years	71.26 ± 8.46	71.10 ± 8.31	0.835
Education, years	8.82 ± 4.53	10.90 ± 3.84	**<0.001**
MMSE	15.72 ± 6.28	28.81 ± 1.26	**<0.001**
Female, *n* (%)	137 (55.69)	137 (56.15)	0.919
*APOE* genotype, *n* (%)			
ε2/ε2	2 (0.81)	2 (0.82)	
ε2/ε3	20 (8.13)	28 (11.48)	
ε2/ε4	4 (1.63)	5 (2.05)	
ε3/ε3	98 (39.84)	160 (65.57)	
ε3/ε4	90 (36.59)	49 (20.08)	
ε4/ε4	32 (13.01)	0 (0)	
*APOE* ε4 carriers, *n* (%)	126 (51.22)	54 (22.13)	**<0.001**

*AD, Alzheimer’s Disease; APOE, Apolipoprotein E; MMSE, Mini Mental Status Evaluation. Demographic data were measured using non-parametric Mann-Whitney U-test for skewed distribution variable. For gender, genotype distribution and allele frequencies, values were expressed as number (%) using chi-square test. Bold values denote significant difference.*

### DNA Extraction and Genotype Analysis

The genomic DNA was extracted from blood (2 ml) stored in an ethylene diamine tetraacetie acid (EDTA) anticoagulation tube using the phenol-chloroform-isopropyl alcohol method. Polymerase chain reaction (PCR) and extension primers scheme were achieved through Primer 5 software (PREMIER Biosoft International, Version 5.00). PCR materials were conducted by purification with phosphorylase (FastAP, Applied Biosystems) and exonuclease I (EXO I, Applied Biosystems). A consequent extension was applied by the ABI SNaPshot Multiplex Kit (Applied Biosystems). Extended products were purified with FastAP and loaded into ABI3730xl (Applied Biosystems). GeneMapper 4.0 (Applied Biosystems) was used to conduct data analysis. The SNaPshot technique (Applied Biosystems) was utilized for genotyping of SNPs. The following SNPs were tested: rs10792832, rs11136000, rs11218343, rs1990620, rs1990622, rs3173615, rs34860942, rs3764650, rs3792646, rs3818361, rs3851179, rs4147929, rs56081887, rs5848, rs6656401, rs6701713, rs6733839, rs704180, rs744373, rs9331888, and rs9637454. The SNPs rs429358 and rs7412 of the *APOE* gene were determined by Sanger sequencing. Details of primers were described in Supplementary Table 1.

### Statistical Analysis

Statistical evaluations involved in the paper were conducted using SPSS software (two-sided significance level: *P*-value < 0.05) (version 26.0; IBM SPSS) or PLINK (Purcell et al., 2007) version 1.9.^1^ The SNP with minor allele frequencies (MAF) < 0.01, call rate < 95%, or not in Hardy-Weinberg equilibrium (HWE) were excluded. Differences in age, education level, and MMSE score between the two groups were examined for continuous variables using the Student’s *t*-test or non-parametric Mann-Whitney *U*-test. Dichotomous variables (such as gender, genotype distribution, allele frequency, and HWE) evaluations were conducted by the chi-square test. Then, logistic regression analysis adjusting for age and gender was used to figure out the odds ratio (OR) and 95% confidence intervals (CI) under varied genetic models (allele, dominant, recessive, and additive). It was defined that “A” means the major allele and “a” is the minor allele in the paper. We also defined dominant as 1 (aa + Aa) vs. 0 (AA), recessive as 1 (aa) vs. 0 (AA + Aa), and additive as 0 (AA) vs. 1 (Aa) vs. 2 (aa). Furthermore, the Bonferroni correction method was applied to perform multiple tests with aim to make the conclusion statistically convincing. G*Power software was used to evaluate all the SNPs’ genetic power.

## Results

### Demographic Characteristics of the Subjects

A total of 246 patients with AD and 244 age- and gender-matched healthy controls were enrolled. No statistically significant difference was found in age and gender between AD and the control group (all *P* > 0.05). In addition, patients with AD were less educated in comparison to the control group (*P* < 0.001), which matched well with the previous studies (Xu et al., 2016; Larsson et al., 2017). Patients with AD showed statistically significant lower MMSE scores compared with healthy controls (*P* < 0.001). Moreover, the patients with AD showed a much higher proportion of *APOE* ε4 allele carriers than the control subjects (*P* < 0.001). Detailed information about the participants was summarized in Table 1.

### Association Analysis of Single Nucleotide Polymorphisms and Alzheimer’s Disease in Various Genetic Models

The genotype distribution of all SNPs in AD and controls was consistent with HWE. The minimum allele and genotype frequencies for all related SNPs were shown in Supplementary Table 2. Significant statistical differences were noted in the allele frequencies of rs3764650 (*P* = 0.010) and rs4147929 (*P* = 0.006) between patients with AD and the healthy ones. Regarding SNP genotype frequencies, it was detected that at rs3764650, the genotypes TT and GT experienced a higher risk for AD than the genotype GG, while at rs4147929, the genotypes GG and AG experienced a higher risk of AD than the genotype AA. Also, statistically significant difference also existed between patients with AD and controls of genotype frequencies in rs3764650 (*P* = 0.015) and rs4147929 (*P* = 0.030). The higher *APOE* level enhanced the risk of developing AD as shown in Table 1. Hence, these data were stratified by *APOE* ε4 levels to determine whether they impacted the correlation between SNPs and AD susceptibility. Among *APOE* ε4 carriers, the allele and genotype frequencies of *ABCA7* rs3764650 were substantially different between AD and control cases [allele: *P* = 0.030, odds ratio (OR) = 1.80, 95% confidence interval (CI) 1.05–3.06, genotype: *P* = 0.034], and allele G was higher in the AD group than that in the control group (Table 2A). After adjusting for age and gender, the dominant and additive models of rs3764650 were nominally significantly related to AD (dominant model: *P* = 0.016, OR = 2.32, 95% CI 1.17–4.59; additive model: *P* = 0.043, OR = 1.77, 95% CI 1.02–3.08) (Table 2A). However, after the Bonferroni correction, these associations did not persist. Moreover, among *APOE* ε4 carriers, the allele and genotype frequencies of *ABCA7* rs4147929 showed the obvious difference between patients with AD and healthy controls (allele: *P* = 0.001, OR = 2.49, 95% CI 1.42–4.37, genotype: *P* = 0.003), with allele A higher in the case group than that in the control group (Table 2B). After adjusting for age and gender, the dominant and additive models of rs4147929 were related to AD (dominant model: *P* = 0.002, OR = 3.06, 95% CI 1.52–6.17; additive model: *P* = 0.004, OR = 2.33, 95% CI 1.31–4.13) (Table 2B). Bonferroni correction was needed to confirm the relationship. To further examine the *ABCA7* genetic association of AD, four genetic models including allele, dominant, recessive, and additive models were analyzed by logistic regression (Table 3 and Supplementary Table 3). The results revealed that rs3764650 of *ABCA7* was correlated with the AD morbidity in the allele model (*P* = 0.010, OR = 1.43, 95% CI 1.09–1.89) without adjusting the age and gender, dominant (*P* = 0.004, OR = 1.71, 95% CI 1.19–2.46), and additive (*P* = 0.012, OR = 1.42, 95% CI 1.08–1.86) models after adjusting the age and gender. In addition, rs4147929 of *ABCA7* was correlated with the risk of developing AD in the allele (*P* = 0.006, OR = 1.45, 95% CI 1.11–1.89) without adjusting the age and gender, dominant (*P* = 0.012, OR = 1.59, 95% CI 1.11–2.27) and additive (*P* = 0.010, OR = 1.40, 95% CI 1.08–1.81) models after adjusting the age and gender. Again, the conclusion also needs to be confirmed by Bonferroni correction.

**TABLE 2A:** Association of rs3764650 of *ABCA7* gene with AD risk stratified by *APOE* ε4 status.

rs3764650	MAF (AD/control)	Allele		Genotype	Dominant model (adjusted)		Recessive model (adjusted)		Additive model (adjusted)	
		*P*-value	OR (95% CI)	*P*-value	*P*-value	OR (95% CI)	*P*-value	OR (95% CI)	*P*-value	OR (95% CI)
Total	0.356/0.278	0.010	1.43 (1.09–1.89)	0.015	0.004	1.71 (1.19–2.46)	0.419	1.27 (0.71–2.26)	0.012	1.42 (1.08–1.86)
*APOE* ε4(+)	0.349/0.230	**0.030**	1.80 (1.05–3.06)	**0.034**	**0.016**	2.32 (1.17–4.59)	0.709	1.26 (0.38–4.13)	**0.043**	1.77 (1.02–3.08)
*APOE* ε4(-)	0.363/0.291	0.066	1.38 (0.98–1.96)	0.187	0.083	1.51 (0.95–2.42)	0.335	1.42 (0.70–2.87)	0.085	1.35 (0.96–1.89)

**TABLE 2B:** Association of rs4147929 of *ABCA7* gene with AD risk stratified by *APOE* ε4 status.

rs4147929	MAF (AD/control)	Allele		Genotype	Dominant model (adjusted)		Recessive model (adjusted)		Additive model (adjusted)	
		*P*-value	OR (95% CI)	*P*-value	*P*-value	OR (95% CI)	*P*-value	OR (95% CI)	*P*-value	OR (95% CI)
Total	0.382/0.299	0.006	1.45 (1.11–1.89)	0.030	0.012	1.59 (1.11–2.27)	0.101	1.54 (0.92–2.59)	0.010	1.40 (1.08–1.81)
*APOE* ε4(+)	0.369/0.190	**0.001**	2.49 (1.42–4.37)	**0.003**	**0.002**	3.06 (1.52–6.17)	0.200	2.32 (0.64–8.36)	**0.004**	2.33 (1.31–4.13)
*APOE* ε4(-)	0.396/0.327	0.083	1.35 (0.96–1.89)	0.236	0.290	1.29 (0.81–2.06)	0.119	1.64 (0.88–3.07)	0.129	1.28 (0.93–1.77)

*AD, Alzheimer’s Disease; Allele, minor allele; CI, confidence interval; MAF, minor allele frequency; OR, odds ratio. Dominant was defined as 1 (aa + Aa) vs. 0 (AA), recessive was defined as 1 (aa) vs. 0 (AA + Aa), and additive was defined as 0 (AA) vs. 1 (Aa) vs. 2 (aa). (A: major allele, a: minor allele). P allele and P genotype was examined using Chi-square test. P-value was adjusted for age and gender by logistic regression analysis. Bold values were indicated the significant results.*

**TABLE 3:** Association of SNPs of candidate genes and odds ratio to AD risk.

Gene	SNP	Minor allele	Allele model			Dominant model (Adjusted)			Recessive model (Adjusted)			Additive model (Adjusted)		
						
			*P-*value	OR	95% CI	*P*-value	OR	95% CI	*P*-value	OR	95% CI	*P*-value	OR	95% CI
*ABCA7*	rs3764650	**G**	**0.010**	1.43	1.09–1.89	**0.004**	1.71	1.19–2.46	0.419	1.27	0.71–2.26	**0.012**	1.42	1.08–1.86
*ABCA7*	rs4147929	**A**	**0.006**	1.45	1.11–1.89	**0.012**	1.59	1.11–2.27	0.101	1.54	0.92–2.59	**0.010**	1.40	1.08–1.81

*AD, Alzheimer’s Disease; CI, confidence interval; OR, odds ratio; SNP, single nucleotide polymorphism. P allele was examined using the Chi-square test. P-value was adjusted for age and gender with logistic regression. Bold indicates statistically significant values.*

## Discussion

The interaction between rs3764650 and rs4147929 of *ABCA7* and the risk factors of AD in southern China was validated in the paper. Differences among *APOE* ε4 carriers were more dominant when allele and genotype distributions were stratified by *APOE* ε4 status. It was found that individuals with a heterozygote GT at rs3764650 had a higher susceptibility to AD, while individuals with heterozygote AG at rs4147929 suffered higher AD morbidity. But the relationship between the remaining SNPs and AD was not replicated in the southern Chinese group. Similarly, varied populations could suffer different AD risk genetic variants. Last but not least, the relatively limited sample compared to recent consortium-based GWAS may lead to the dismissal of replication.

A highly conserved protein, a part of the ABCB family of ATP-binding cassette (ABC) transporters, was encoded by the *ABCA7*, which was known as ATP-binding cassette subfamily A member 7 (Takahashi et al., 2005). The *ABCA7* also plays a role in the transportation of cholesterol across membranes (Le Guennec et al., 2016), particularly in the hippocampal CA1 neurons and microglia (Kim et al., 2005), suggesting a role in amyloid clearance and fibril formation (Chan et al., 2008; Aikawa et al., 2018). Recently, some studies investigated the interaction between *ABCA7* rs3764650 polymorphism and the morbidity of AD. However, the results were controversial (Hollingworth et al., 2011; Allen et al., 2012; Lambert et al., 2013; Reitz et al., 2013). Our study confirmed that *ABCA7* rs3764650 might be important genetic factor in the pathophysiology of AD. Furthermore, previous researchers found that the *ABCA7* rs4147929 might be a predisposing factor for late-onset AD (Hollingworth et al., 2011; Lambert et al., 2013; Talebi et al., 2020). In line with that, this study provides supportive evidence for the relationship between rs4147929 and AD. However, it should be noted that the conclusion needs further verification. Meanwhile, the shortcoming of our study is that there is no distinction between early-onset and late-onset AD, and further subgroup research stratified by age of onset will be carried out in the future.

The research plays an important role in figuring out the basic genes of AD in the Asian population and offers useful findings that genetic risk factors varied in different populations. It should be noted that there are certain limitations in the study. Most importantly, the relatively small-sized sample due to its single-centered nature may affect the validity. A consequent meta-analysis of the Asian population with a bigger sample size should be conducted. Secondly, follow-up studies are required to evaluate more participants’ loci for AD susceptibility. Simultaneously, the participants involved here should be followed regularly to find their cognitive variations and figure out the association between genetic polymorphisms and clinical performance. Last but not least, we will conduct a comprehensive neuropsychological battery to assess cognitive function in the future study.

## Conclusion

In summary, this study suggested that the G-allele at rs3764650 and the A-allele at rs4147929 appeared at higher risk for developing AD in the southern Chinese population, particularly in *APOE* ε4 carriers. In addition, rs3764650 and rs4147929 of *ABCA7* were observed to be associated with AD. Further investigations on the role of the risk genes in the pathogenesis of AD are essential in the following research.

## Data Availability Statement

The original contributions presented in the study are included in the article/Supplementary Material, further inquiries can be directed to the corresponding author/s.

## Ethics Statement

The studies involving human participants were reviewed and approved by this research was permitted by the Committee on Medical Ethics of Ruijin Hospital Affiliated to Shanghai Jiao Tong University School of Medicine. The patients/participants provided their written informed consent to participate in this study.

## Author Contributions

JL and WX conceived and designed the studies. LW analyzed the results and wrote the manuscript. LW, YJ, AZ, XX, GY, YZ, YW, and YD performed the research. All authors contributed to the article and approved the submitted version.

## Conflict of Interest

The authors declare that the research was conducted in the absence of any commercial or financial relationships that could be construed as a potential conflict of interest.

## Publisher’s Note

All claims expressed in this article are solely those of the authors and do not necessarily represent those of their affiliated organizations, or those of the publisher, the editors and the reviewers. Any product that may be evaluated in this article, or claim that may be made by its manufacturer, is not guaranteed or endorsed by the publisher.

## Footnotes

1
http://pngu.mgh.harvard.edu/purcell/plink/
