# Supplementary material for: Analysis of Genetic Association Between ABCA7 Polymorphism and Alzheimer’s Disease Risk in the Southern Chinese Population
Source: Front Aging Neurosci. 2022 May 25;14:819499. doi: 10.3389/fnagi.2022.819499 (PMC9175022; doi:10.3389/fnagi.2022.819499)
Supplement: Supplementary file 1 [file Data_Sheet_1.docx]

**Table S1. Primers of tested single nucleotide polymorphisms.**

| SNPs | Forward Primers | Reverse Primers |
| --- | --- | --- |
| rs10792832 | GGAGCAGGACTCAATGAACATC | GGCCATCCTTTCTCACACTTAC |
| rs11136000 | CTGCAGACTCCCTGAATCTTAC | CACCACCACACCAGCT |
| rs11218343 | TCCCAAACTGCTGGGATTAC | AGCCACCACTCATGAGAAAC |
| rs1990620 | AAACATTTGCTCAGCCCATTAG | CAGGGTAAGGAAGATCATGAAGAG |
| rs1990622 | CCCTTTATCTCGCCTACACATT | TTGTACTGCAGCTCTGTTCTC |
| rs3173615 | GTTATTGGAAAGGCACGCTTAAA | CCCACCTCCTTCAAGTTATATGAT |
| rs34860942 | CATCTTGCCTCTTGCTTACCT | CACCAAGCACTTTGCACATATAG |
| rs3764650 | GATGCCCTATCCGTGCTATG | TCTCAAGGCGGGAAGATTTG |
| rs3792646 | GCAAATAAACATGACTGCTTCCT | GGCTTCATATCTGGCCTTAGTT |
| rs3818361 | GTGGCTACTGAACTACCAATCT | GTCCTGATGGCTTAGGGTATG |
| rs3851179 | CAGTCTCTTGCTACCCATGTC | ACCCGCTTCATAGGGTTATTG |
| rs4147929 | CACCACACTATGTCCCATTCTC | CGTACCATGAGAGTCCCAGA |
| rs56081887 | GATTGTGCGAACTTGGGAAAG | CCCTAAGGGTAAACACCAGTATTAG |
| rs5848 | GGTCCAGGGAGAATTTGGTTAG | TGTGAGGGACAGTACTGAAGA |
| rs6656401 | TCCTTCTCTGTCTCCATCTTC | CCTCTAAATCCATGTCCAGTGT |
| rs6701713 | TGTACCACGCGAAAGGAAG | GGAGGTGTTACAGCACACTAAT |
| rs6733839 | CCAGTGACTTACGCTGACTTT | TTCCCGTTCCATCCTGTTTC |
| rs704180 | TCACCTGTCATGTGGCTTAAC | CGTTTAGGATTTGTCATTTACTGAGATG |
| rs744373 | GATCTCACCTTTGACCTGACTAAC | CCAGCCTCCACTGCAAATTA |
| rs9331888 | ATTCCCTTCGGAGAGTAGAGAG | GGAAAGGGTCCATTGTGTAAGA |
| rs9637454 | GACTCCTACCTCTTAACTCAGGT | ATAAGGAACCAGCAGGAGATTG |

*Abbreviations: SNP = single nucleotide polymorphism.*

**Table S2. The alleles and genotype distribution of candidate genes in the controls and AD cases.**

| **SNP** | **Allele [n (%)]** | | ***P value*** | **Genotype [n (%)]** | | | ***P value*** | **HWE *P* value** | **Genetic Power** |
| --- | --- | --- | --- | --- | --- | --- | --- | --- | --- |
|  |  |  | **OR** |  |  |  |  |  |  |
|  |  |  | **95% CI** |  |  |  |  |  |  |
| rs10792832 | A | G | 0.223 | AA | AG | GG | 0.371 | 0.444 | 0.224 |
| AD | 177(0.360) | 315(0.640) | 0.852 | 28(0.114) | 121(0.492) | 97(0.394) |  |  |  |
| Control | 194(0.398) | 294(0.602) | (0.66-1.10) | 38(0.156) | 118(0.484) | 88(0.361) |  |  |  |
| rs11136000 | T | C | 0.886 | TT | TC | CC | 0.511 | 0.758 | 0.164 |
| AD | 86(0.175) | 406(0.825) | 0.976 | 6(0.024) | 74(0.301) | 166(0.675) |  |  |  |
| Control | 87(0.178) | 401(0.822) | (0.70-1.36) | 10(0.041) | 67(0.275) | 167(0.684) |  |  |  |
| rs11218343 | C | T | 0.241 | CC | CT | TT | 0.274 | 0.479 | 0.282 |
| AD | 118(0.240) | 374(0.760) | 0.842 | 13(0.053) | 92(0.374) | 141(0.573) |  |  |  |
| Control | 133(0.273) | 355(0.727) | (0.63-1.12) | 22(0.090) | 89(0.365) | 133(0.545) |  |  |  |
| rs1990620 | A | G | 0.529 | AA | AG | GG | 0.512 | 0.550 | 0.163 |
| AD | 166(0.337) | 326(0.663) | 0.919 | 27(0.110) | 112(0.455) | 107(0.435) |  |  |  |
| Control | 174(0.357) | 314(0.643) | (0.71-1.20) | 35(0.143) | 104(0.426) | 105(0.430) |  |  |  |
| rs1990622 | A | G | 0.445 | AA | AG | GG | 0.618 | 0.620 | 0.129 |
| AD | 167(0.339) | 325(0.661) | 0.903 | 28(0.114) | 111(0.451) | 107(0.435) |  |  |  |
| Control | 177(0.363) | 311(0.637) | (0.69-1.17) | 35(0.143) | 107(0.439) | 102(0.418) |  |  |  |
| rs3173615 | C | G | 0.445 | CC | CG | GG | 0.539 | 0.488 | 0.154 |
| AD | 167(0.339) | 325(0.661) | 0.903 | 28(0.114) | 111(0.451) | 107(0.435) |  |  |  |
| Control | 177(0.363) | 311(0.637) | (0.69-1.17) | 36(0.148) | 105(0.430) | 103(0.422) |  |  |  |
| rs34860942 | G | C | 0.351 | GG | GC | CC | 0.430 | 1.000 | 0.196 |
| AD | 54(0.110) | 438(0.890) | 0.832 | 4(0.016) | 46(0.187) | 196(0.797) |  |  |  |
| Control | 63(0.129) | 425(0.871) | (0.56-1.23) | 3(0.012) | 57(0.234) | 184(0.754) |  |  |  |
| rs3764650 | G | T | **0.010** | GG | GT | TT | **0.015** | 0.343 | 0.742 |
| AD | 175(0.356) | 317(0.644) | 1.434 | 30(0.122) | 115(0.467) | 101(0.411) |  |  |  |
| Control | 129(0.278) | 335(0.722) | (1.09-1.89) | 23(0.099) | 83(0.358) | 126(0.543) |  |  |  |
| rs3792646 | C | A | 0.898 | CC | CA | AA | 0.895 | 0.616 | 0.067 |
| AD | 22(0.045) | 470(0.955) | 1.041 | 0(0.000) | 22(0.089) | 224(0.911) |  |  |  |
| Control | 21(0.043) | 467(0.957) | (0.56-1.92) | 0(0.000) | 21(0.086) | 223(0.914) |  |  |  |
| rs3818361 | A | G | 0.523 | AA | AG | GG | 0.590 | 0.278 | 0.138 |
| AD | 180(0.366) | 312(0.634) | 1.089 | 38(0.154) | 104(0.423) | 104(0.423) |  |  |  |
| Control | 169(0.346) | 319(0.654) | (0.84-1.42) | 30(0.123) | 109(0.447) | 105(0.430) |  |  |  |
| rs3851179 | T | C | 0.223 | TT | TC | CC | 0.371 | 0.444 | 0.224 |
| AD | 177(0.360) | 315(0.640) | 0.852 | 28(0.114) | 121(0.492) | 97(0.394) |  |  |  |
| Control | 194(0.398) | 294(0.602) | (0.66-1.10) | 38(0.156) | 118(0.484) | 88(0.361) |  |  |  |
| rs4147929 | A | G | **0.006** | AA | AG | GG | **0.030** | 0.016 | 0.655 |
| AD | 188(0.382) | 304(0.618) | 1.449 | 41(0.167) | 106(0.431) | 99(0.402) |  |  |  |
| Control | 146(0.299) | 342(0.701) | (1.11-1.89) | 28(0.115) | 90(0.369) | 126(0.516) |  |  |  |
| rs56081887 | G | C | 0.658 | GG | GC | CC | 0.735 | 1.000 | 0.099 |
| AD | 56(0.114) | 436(0.886) | 0.916 | 4(0.016) | 48(0.195) | 194(0.789) |  |  |  |
| Control | 60(0.123) | 428(0.877) | (0.62-1.35) | 3(0.012) | 54(0.221) | 187(0.766) |  |  |  |
| rs5848 | T | C | 0.229 | TT | TC | CC | 0.461 | 0.305 | 0.183 |
| AD | 170(0.346) | 322(0.654) | 1.178 | 26(0.106) | 118(0.480) | 102(0.415) |  |  |  |
| Control | 151(0.309) | 337(0.691) | (0.90-1.54) | 21(0.086) | 109(0.447) | 114(0.467) |  |  |  |
| rs6656401 | A | G | 0.833 | AA | AG | GG | 0.831 | 1.000 | 0.079 |
| AD | 14(0.028) | 478(0.972) | 0.924 | 0(0.000) | 14(0.057) | 232(0.943) |  |  |  |
| Control | 15(0.031) | 473(0.969) | (0.44-1.93) | 0(0.000) | 15(0.061) | 229(0.939) |  |  |  |
| rs6701713 | A | G | 0.523 | AA | AG | GG | 0.590 | 0.278 | 0.138 |
| AD | 180(0.366) | 312(0.634) | 1.089 | 38(0.154) | 104(0.423) | 104(0.423) |  |  |  |
| Control | 169(0.346) | 319(0.654) | (0.84-1.42) | 30(0.123) | 109(0.447) | 105(0.430) |  |  |  |
| rs6733839 | T | C | 0.598 | TT | TC | CC | 0.660 | 0.784 | 0.118 |
| AD | 224(0.455) | 268(0.545) | 1.070 | 48(0.195) | 128(0.520) | 70(0.285) |  |  |  |
| Control | 214(0.439) | 274(0.561) | (0.83-1.38) | 48(0.197) | 118(0.484) | 78(0.320) |  |  |  |
| rs704180 | A | G | 0.869 | AA | AG | GG | 0.953 | 0.250 | 0.057 |
| AD | 185(0.376) | 307(0.624) | 0.978 | 31(0.126) | 123(0.500) | 92(0.374) |  |  |  |
| Control | 186(0.381) | 302(0.619) | (0.76-1.27) | 33(0.135) | 120(0.492) | 91(0.373) |  |  |  |
| rs744373 | G | A | 0.869 | GG | GA | AA | 0.952 | 0.499 | 0.057 |
| AD | 182(0.370) | 310(0.630) | 0.979 | 31(0.126) | 120(0.488) | 95(0.386) |  |  |  |
| Control | 183(0.375) | 305(0.625) | (0.76-1.27) | 33(0.135) | 117(0.480) | 94(0.385) |  |  |  |
| rs9331888 | G | C | 0.744 | GG | GC | CC | 0.438 | 0.717 | 0.193 |
| AD | 236(0.480) | 256(0.520) | 1.043 | 52(0.211) | 132(0.537) | 62(0.252) |  |  |  |
| Control | 229(0.469) | 259(0.531) | (0.81-1.34) | 56(0.230) | 117(0.480) | 71(0.291) |  |  |  |
| rs9637454 | G | A | 0.848 | GG | GA | AA | 0.758 | 0.086 | 0.094 |
| AD | 247(0.502) | 245(0.498) | 1.025 | 55(0.224) | 137(0.557) | 54(0.220) |  |  |  |
| Control | 242(0.496) | 246(0.504) | (0.80-1.32) | 57(0.234) | 128(0.525) | 59(0.242) |  |  |  |

*Abbreviations: AD = Alzheimer’s Disease, CI = confidence interval, HWE = Hardy-Weinberg equilibrium, OR = odds ratio, SNP = single nucleotide polymorphism. HWE was calculated by chi-square test. Bold indicates statistically significant values.*

**Table S3. Association of SNP of candidate genes with AD risk in four genetic models.**

| **Candidate Gene** | **SNP** | **MAF (AD/Control)** | **Allele model** | | | **Dominant model (adjusted)** | | |
| --- | --- | --- | --- | --- | --- | --- | --- | --- |
|  |  |  | ***P* value** | **OR** | **95% CI** | ***P value*** | **OR** | **95% CI** |
| *PICALM* | rs10792832 | 0.360/0.398 | 0.223 | 0.85 | 0.66-1.10 | 0.430 | 0.86 | 0.60-1.25 |
| *CLU* | rs11136000 | 0.175/0.178 | 0.886 | 0.98 | 0.70-1.36 | 0.829 | 1.04 | 0.71-1.53 |
| *SORL1* | rs11218343 | 0.240/0.273 | 0.241 | 0.84 | 0.63-1.12 | 0.531 | 0.89 | 0.62-1.28 |
| *TMEM106B* | rs1990620 | 0.337/0.357 | 0.529 | 0.92 | 0.71-1.20 | 0.922 | 0.98 | 0.69-1.41 |
| *TMEM106B* | rs1990622 | 0.339/0.363 | 0.445 | 0.90 | 0.69-1.17 | 0.708 | 0.93 | 0.65-1.34 |
| *TMEM106B* | rs3173615 | 0.339/0.363 | 0.445 | 0.90 | 0.69-1.17 | 0.777 | 0.95 | 0.66-1.36 |
| *RBFOX1* | rs34860942 | 0.110/0.129 | 0.351 | 0.83 | 0.56-1.23 | 0.260 | 0.78 | 0.51-1.20 |
| *ABCA7* | rs3764650 | 0.356/0.278 | **0.010** | 1.43 | 1.09-1.89 | **0.004** | 1.71 | 1.19-2.46 |
| *C7* | rs3792646 | 0.045/0.043 | 0.898 | 1.04 | 0.56-1.92 | 0.895 | 1.04 | 0.56-1.95 |
| *CR1* | rs3818361 | 0.366/0.346 | 0.523 | 1.09 | 0.84-1.42 | 0.861 | 1.03 | 0.72-1.48 |
| *PICALM* | rs3851179 | 0.360/0.398 | 0.223 | 0.85 | 0.66-1.10 | 0.430 | 0.86 | 0.60-1.25 |
| *ABCA7* | rs4147929 | 0.382/0.299 | **0.006** | 1.45 | 1.11-1.89 | **0.012** | 1.59 | 1.11-2.27 |
| *RBFOX1* | rs56081887 | 0.114/0.123 | 0.658 | 0.92 | 0.62-1.35 | 0.552 | 0.88 | 0.57-1.35 |
| *GRN* | rs5848 | 0.346/0.309 | 0.229 | 1.18 | 0.90-1.54 | 0.237 | 1.24 | 0.87-1.78 |
| *CR1* | rs6656401 | 0.028/0.031 | 0.833 | 0.92 | 0.44-1.93 | 0.832 | 0.92 | 0.44-1.96 |
| *CR1* | rs6701713 | 0.366/0.346 | 0.523 | 1.09 | 0.84-1.42 | 0.861 | 1.03 | 0.72-1.48 |
| *BIN1* | rs6733839 | 0.455/0.439 | 0.598 | 1.07 | 0.83-1.38 | 0.389 | 1.19 | 0.80-1.75 |
| *ABCC9* | rs704180 | 0.376/0.381 | 0.869 | 0.98 | 0.76-1.27 | 0.971 | 0.99 | 0.69-1.43 |
| *BIN1* | rs744373 | 0.370/0.375 | 0.869 | 0.98 | 0.76-1.27 | 0.992 | 1.00 | 0.69-1.44 |
| *CLU* | rs9331888 | 0.480/0.470 | 0.744 | 1.04 | 0.81-1.34 | 0.327 | 1.22 | 0.82-1.82 |
| *KCNMB2* | rs9637454 | 0.502/0.496 | 0.848 | 1.03 | 0.80-1.32 | 0.575 | 1.13 | 0.74-1.73 |
| **Candidate Gene** | **SNP** | **MAF (AD/Control)** | **Recessive Model (adjusted)** | | | **Additive Model (adjusted)** | | |
|  |  |  | ***P value*** | **OR** | **95% CI** | ***P value*** | **OR** | **95% CI** |
| *PICALM* | rs10792832 | 0.360/0.398 | 0.179 | 0.70 | 0.41-1.18 | 0.211 | 0.84 | 0.65-1.10 |
| *CLU* | rs11136000 | 0.175/0.178 | 0.293 | 0.57 | 0.20-1.62 | 0.871 | 0.97 | 0.70-1.35 |
| *SORL1* | rs11218343 | 0.240/0.273 | 0.112 | 0.56 | 0.28-1.14 | 0.248 | 0.85 | 0.64-1.12 |
| *TMEM106B* | rs1990620 | 0.337/0.357 | 0.263 | 0.74 | 0.43-1.26 | 0.536 | 0.92 | 0.71-1.20 |
| *TMEM106B* | rs1990622 | 0.339/0.363 | 0.327 | 0.77 | 0.45-1.31 | 0.452 | 0.91 | 0.70-1.17 |
| *TMEM106B* | rs3173615 | 0.339/0.363 | 0.267 | 0.74 | 0.44-1.26 | 0.452 | 0.91 | 0.70-1.17 |
| *RBFOX1* | rs34860942 | 0.110/0.129 | 0.728 | 1.31 | 0.29-5.98 | 0.350 | 0.83 | 0.56-1.23 |
| *ABCA7* | rs3764650 | 0.356/0.278 | 0.419 | 1.27 | 0.71-2.26 | **0.012** | 1.42 | 1.08-1.86 |
| *C7* | rs3792646 | 0.045/0.043 | NA | NA | NA | 0.895 | 1.04 | 0.56-1.95 |
| *CR1* | rs3818361 | 0.366/0.346 | 0.311 | 1.31 | 0.78-2.19 | 0.529 | 1.09 | 0.84-1.40 |
| *PICALM* | rs3851179 | 0.360/0.398 | 0.179 | 0.70 | 0.41-1.18 | 0.211 | 0.84 | 0.65-1.10 |
| *ABCA7* | rs4147929 | 0.382/0.299 | 0.101 | 1.54 | 0.92-2.59 | **0.010** | 1.40 | 1.08-1.81 |
| *RBFOX1* | rs56081887 | 0.114/0.123 | 0.728 | 1.31 | 0.29-5.98 | 0.652 | 0.91 | 0.62-1.35 |
| *GRN* | rs5848 | 0.346/0.309 | 0.450 | 1.26 | 0.69-2.32 | 0.210 | 1.19 | 0.91-1.57 |
| *CR1* | rs6656401 | 0.028/0.031 | NA | NA | NA | 0.832 | 0.92 | 0.44-1.96 |
| *CR1* | rs6701713 | 0.366/0.346 | 0.311 | 1.31 | 0.78-2.19 | 0.529 | 1.09 | 0.84-1.40 |
| *BIN1* | rs6733839 | 0.455/0.439 | 0.962 | 0.99 | 0.63-1.55 | 0.590 | 1.07 | 0.83-1.38 |
| *ABCC9* | rs704180 | 0.376/0.381 | 0.762 | 0.92 | 0.55-1.56 | 0.857 | 0.98 | 0.75-1.27 |
| *BIN1* | rs744373 | 0.370/0.375 | 0.761 | 0.92 | 0.54-1.56 | 0.872 | 0.98 | 0.75-1.27 |
| *CLU* | rs9331888 | 0.480/0.469 | 0.631 | 0.90 | 0.59-1.38 | 0.735 | 1.05 | 0.81-1.35 |
| *KCNMB2* | rs9637454 | 0.502/0.496 | 0.786 | 0.94 | 0.62-1.44 | 0.859 | 1.02 | 0.79-1.33 |

*Abbreviations: AD = Alzheimer’s Disease, CI = confidence interval, OR = odds ratio, SNP = single nucleotide polymorphism. P value was adjusted for age and gender. Bold indicates statistically significant values.*
